# Plasmonic/Magnetic Multifunctional nanoplatform for Cancer Theranostics

**DOI:** 10.1038/srep34874

**Published:** 2016-10-10

**Authors:** M. Ravichandran, Goldie Oza, S. Velumani, Jose Tapia Ramirez, Francisco Garcia-Sierra, Norma Barragan Andrade, A. Vera, L. Leija, Marco A. Garza-Navarro

**Affiliations:** 1 Program on Nanoscience and Nanotechnology, Av. 2508 National Polytechnic Institute, Gustavo A. Madero, San Pedro Zacatenco, 07360 Mexico City, Mexico; 2 Department of Genetics and Molecular Biology, Av. 2508 National Polytechnic Institute, Gustavo A. Madero, San Pedro Zacatenco, 07360 Mexico City, Mexico; 3 Department of Electrical Engineering, Av. 2508 National Polytechnic Institute, Gustavo A. Madero, San Pedro Zacatenco, 07360 Mexico City, Mexico; 4 Department of Cell Biology, Av. 2508 National Polytechnic Institute, Gustavo A. Madero, San Pedro Zacatenco, 07360 Mexico City, Mexico; 5 Department of Electrical Engineering - Bioelectronics Section, CINVESTAV-IPN, Av. 2508 National Polytechnic Institute, Gustavo A. Madero, San Pedro Zacatenco, 07360 Mexico City; 6 Department of Mechanical and Electrical Engineering, Universidad Autonoma de Nuevo Leon, San Nicolás de Los Garza, Nuevo León, 66451 Mexico City, Mexico.

## Abstract

A multifunctional magneto-plasmonic CoFe_2_O_4_@Au core-shell nanoparticle was developed by iterative-seeding based method. This nanocargo consists of a cobalt ferrite kernel as a core (Nk) and multiple layers of gold as a functionalizable active stratum, (named as Nk@A after fifth iteration). Nk@A helps in augmenting the physiological stability and enhancing surface plasmon resonance (SPR) property. The targeted delivery of Doxorubicin using Nk@A as a nanopayload is demonstrated in this report. The drug release profile followed first order rate kinetics optimally at pH 5.4, which is considered as an endosomal pH of cells. The cellular MR imaging showed that Nk@A is an efficient T_2_ contrast agent for both L6 (r_2_-118.08 mM^−1^s^−1^) and Hep2 (r_2_-217.24 mM^−1^s^−1^) cells. Microwave based magnetic hyperthermia studies exhibited an augmentation in the temperature due to the transformation of radiation energy into heat at 2.45 GHz. There was an enhancement in cancer cell cytotoxicity when hyperthermia combined with chemotherapy. Hence, this single nanoplatform can deliver 3-pronged theranostic applications viz., targeted drug-delivery, T_2_ MR imaging and hyperthermia.

Cancer is the second leading disease which causes major mortality and morbidity worldwide^1^. In cancer therapy, it is crucial to increase the drug specificity and drug efficacy to minimise or completely eradicate significant side-effects on patients^2^. Cancer nanotherapeutics overcome many serious drawbacks of chemotherapy such as non-specific targeting, lower efficacy, insolubility of drug moieties in water and oral bioavailability^3^. Accordingly, Superparamagnetic Iron Oxide Nanoparticles (**SPIONs**) are exploited as an important nanomaterial for cancer detection as well as therapeutics^4^. Such magnetic nanoparticles (**NPs**) gained its momentum because of their single-domain ordering along with their large surface to volume ratio (providing large surface area for attachment of biological entities). Hence, this property makes them a suitable candidate as a contrast agent, drug-carrying cargo and hyperthermal agent^5^. The doping of **SPIONs** with cobalt ions further enhances their magnetic property, thus forming CoFe_2_O_4_ nanokernels (**Nks**). These spinel ferrite **Nks** possess ca. 20–30 times higher magneto-crystalline anisotropy as compared to **SPIONs**; this increases the performance of materials for biomedical applications^6^,^7^,^8^. Specifically, these **Nks** are mostly used in biomedicine than any other spinel structure because of their enhanced magnetic property and large anisotropy^9^. The increased superparamagnetism makes them an efficient system for theranostics^10^,^11^,^12^.

Such superparamagnetic **Nks** are reactive and toxic to cells; hence, gold **NPs** are used for creating a shell on the magnetic core. This architecture is biocompatible and chemically inert in the physiological system^13^. The core-shell nanoparticles (**CSNPs**) possesses unique optical and magnetic properties, thus creating an efficient platform for nanomedicine^14^. The significant benefit of the gold nanoshell is to provide complete protection to the inner magnetic core from a plethora of environmental factors^15^. This coat also acts as an excellent platform for surface modifications^16^,^17^, real-time imaging and drug carrying cargos^18^,^19^.

The major hurdle in synthesising **CSNPs** is that there is no uniform coating of gold shell on the surface of the iron oxide core, even though the ratio between iron and gold is 1:7^20^,^21^. Therefore, gold iteration is a method that improves the formation of **CSNPs** and controls precisely the thickness of Au shell^22^,^23^ on the magnetic core. Even though iron oxide and gold **CSNPs** have been explored extensively^24^,^25^ for more than two decades, there are very few reports about CoFe_2_O_4_@Au nanoparticles^21^,^26^.

Doxorubicin **(Dox)** is one of the potential and most widely used anti-cancer agents for various types of cancers. This drug has shown inimical side effects on healthy cells such as cardiotoxicity, mucositis and myelosuppression^27^,^28^,^29^. These adverse effects are minimised by targeted drug delivery which uses specific molecules such as folic acid **(FA)** since cancerous cells overexpress folate receptors on their surface^30^. Apart from synaphic delivery of drugs, the most crucial parameter is its actual release for killing the cancerous cells. The effective release is dependent on different types of stimuli such as internal (alterations in pH, temperature, redox condition as well as the enzyme activities) or external (such as a magnetic field, radiations and ultrasound)^31^.

Hyperthermia induced by external magnetic field is the most celebrated mechanism that enhances drug release efficiency of the system and are easiest to be used^32^. The synergistic action of hyperthermia and chemotherapy induces apoptosis as well as necrosis in the cancer cells followed by an enhanced immune response. There is a huge impact of hyperthermia-combined chemotherapy on the immune system of hosts since they induce both adaptive as well as innate immunity. Hence, thermo-chemosenitization is considered as the future of clinical research^33^.

This work reports multiple iterative gold seed coated cobalt iron oxide nanokernels (**Nk@A**) as a tri-pronged cancer theranostic agent (Fig. 1). The attachment of **FA** on the surface of **Nk@A** was used for tethering folate receptors present on cancerous cells^30^. Furthermore, **Dox** moieties orchestrated on **FA** attached **Nk@A** were responsible for their anti-cancer activity. Hence, these nanocargos act as proficient drug delivery missiles that targets cancer cells. The drug release profiles were studied using release kinetic models. Moreover, these **Nk@A** also served as a T_2_ contrast agent for MR imaging. Finally, such a complex nanocargo was exploited for microwave based localized hyperthermia of cancer cells.

## Results and Discussions

### Characterization of Plasmonic/Magnetic NPs

**Figure 2:**
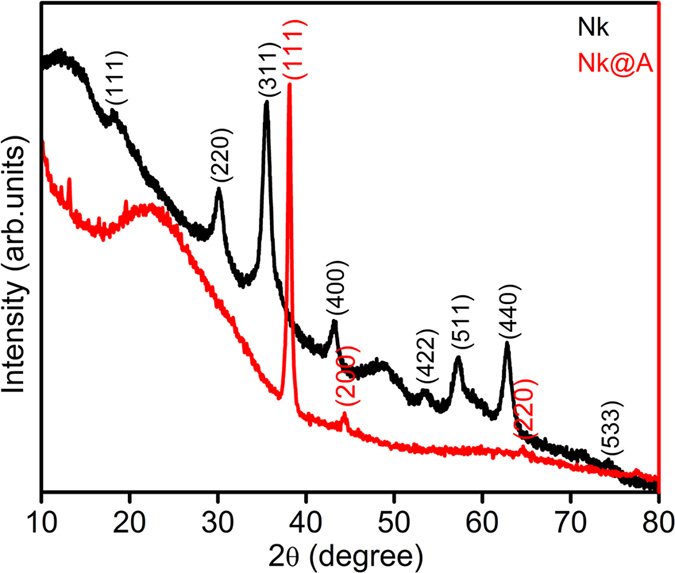
XRD spectrum representing the formation of **Nk** and **Nk@A**.

XRD analysis was carried out to detect the purity and phase crystallinity of the synthesized **Nk** and **Nk@A** (Fig. 2). XRD pattern of **Nk** cubic spinel phase exhibited well-defined diffraction peaks that match with the CoFe_2_O_4_ structures (JCPDS card no. 22-1086)^34^. XRD pattern of **Nk@A** showed reflections that corelated well with the FCC ordering of Au (JCPDS card no. 04-0784), thus confirming the formation of a nanoshell onto **Nk**. In this case, only Au diffraction peaks were observed due to the heavy metal atom effect of Au^18^,^32^. The average particle sizes (D_xrd_) of **Nk** and **Nk@A** were calculated by considering the most intense peaks [**Nk** (220), (311), (440) and **Nk@A** (111)]. According to Scherrer equation, the average crystalline size calculated for **Nk** was 9.68 nm and for **Nk@A**, 16.69 nm. The particle sizes obtained were well corroborated with corresponding TEM images.

**Figure 3:**
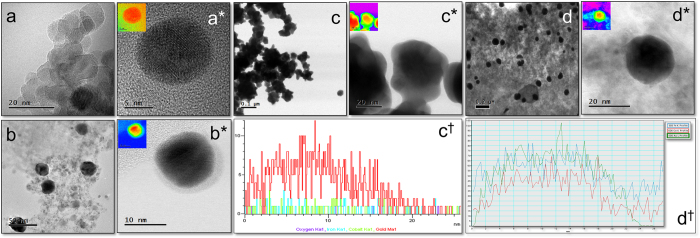
(a(a*)) TEM, HRTEM image of **Nk**, TEM and HAADF-STEM image of: (**b**(b*)) 1^st^ iteration consisting of very thin Au shell and depicting 2 different contrast which proves the formation of core-shell nanoparticles along CTAB layer, (**c**(c*)) 3rd iteration showing nanoflower formation, (**d**(d*)) 5^th^ iterative **Nk@A** with thick Au shell around 6–7 nm (Inset representing the colour mapping of corresponding images), (**c**^**†**^,**d**^**†**^) Line scan analysis showing the distribution of Fe, Co and Au elements from a single nanoparticle.

Figure 3a–d^†^ shows TEM, HR-TEM, HAADF-STEM images and line scan of **Nk** and **Nk@A** (1^st^, 3^rd^ & 5^th^ iterations). TEM image of **Nks** were spherical in shape with the size range of 11–14 nm exhibiting high crystallinity with aggregation which is illustrated in Fig. 3a(a*). The crystal lattice structure of **Nks** were distorted on the surface due to the curvature effect. Hence, the gold shell could grow epitaxially on the surface of **Nks** due to a large lattice mismatch. This leads to the formation of **Nk** as a core and gold as a shell. As iteration increased, the size of the core-shell is also increased five times from 1^st^ to 5^th^ iteration with Au nanoshell. This increment was due to the continued conjugation of nanogold onto the surface of the core **Nk**, which lead to the formation of **Nk@A**. HAADF-STEM analysis clearly demonstrated the discrimination between the core and the shell. This is because this contrast is directly proportional to atomic number (Z). In the 1^st^ iterative step, the **Nk** was coated with Au nanoshell of around 1–1.5 nm (Fig. 3b(b*)) without any aggregation. This proves that the Au iterations not only forms a shell but also stabilizes the nanoparticles. However, in order to enhance the **SPR** property, the iteration was continued; thus leading to the formation of a nanoflower, that constituted of the collective core madeof **Nk** encapsulated by a thick Au shell. The nanocluster showed jagged-like morphology due to non-homogeneously aggregated **Nk**, which leads to highly asymmetric coating of Au layer (Fig. 3c(c*))^35^. The thick gold nanoshell formation after multiple iterations in the **Nk@A** solution could be inferred from the colour change (Figure S1a in the †ESI). Figure 3c† illustrates the line scan analysis of 3^rd^ iterated nanoflowers showing the elemental distribution of Co, Fe and Au in a single nanoparticle. The line scan confirms that Au signal is seen on the surface of **Nks** and Co, Fe signals are enriched in the inner core of the **Nks**. But as the iterations (5^th^ iterations) continued, the Au seeds started to fill in the empty space of the knobby structures^22^. This resulted in the formation of separated spherical **Nk@A** and consecutively the shell size increased to 5–6 nm (Fig. 3d(d*)). Similar kind of **NPs** were obtained for Au@Fe_3_O_4_, which had a thin shell of Au^36^. Figure 3d† represents the corresponding line scan, which clearly shows the Co K, Fe K edges in the core and Au L edges in the shell similar to that of 3^rd^ iterated particles. Additionally to the line scan intensities illustrated in Fig. 3c†,d†, it is interesting to define the stoichiometry represented by the Au Lα, whose ratio is higher than the core elements such as Co and Fe Kα. This proves that the Au signal is exhibited strongly than the signal from the core. Therefore, these results suggest that the formation of **CSNPs** expressing typical elemental composition of high Au content than the core elements, is well corroborated from XRD spectra. The colour mapping shown in Fig. 3(a* proves that the nanostructures are made up of two different metals depending on the electronic density of the atoms. This colour mapping was carried out using the Digital Micrograph 3.7.0 by Gatan software. EDS spectra was carried out to determine the composition of **CSNPs** for the 1^st^, 3^rd^ and 5^th^ iterations (Figure S1b,c,d in the †ESI) showing the signal of Au, Co & Fe^37^. The spectra clearly distinguishes the different Au iterations from 1 to 5 just by increasing order of Au signal intensity.

**Figure 4:**
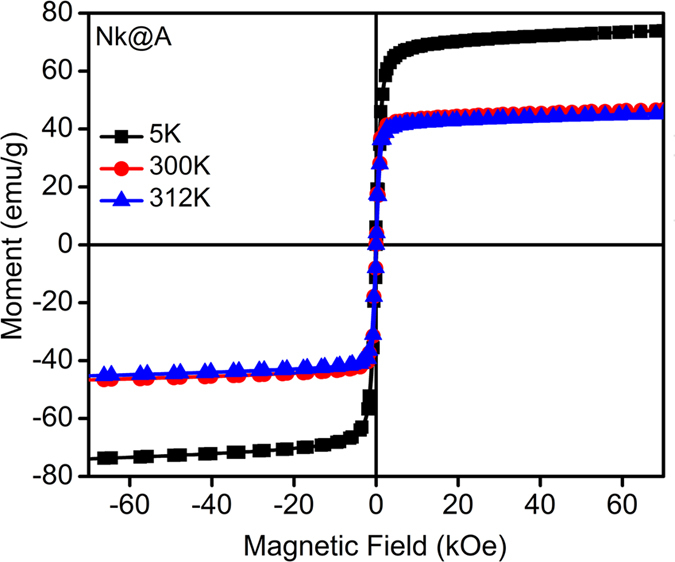
Magnetic measurements for **Nk@A** using SQUID at 5 K, 300 K & 312 K.

The magnetic property of **Nk@A** is imperative to have an effective penetration in the cancer cell^38^. SQUID analysis (Fig. 4) shows a decrement in magnetic saturation (MS) from 74 to 45 emu/g, along with the diminishing hysteretic features. As temperature increased from 5 to 312 K, coercivity (HC) and remanence decreased from 146 to 32Oe and from 8 to 2 emu/g, respectively (Table S1 in the †ESI).

This magnetic behaviour was attributed to the thermal relaxation of the magnetic moments of the **Nk@A**. This behaviour was also ascribed to the re-orientation of the magnetic moments of **NPs** caused by the thermal energy once it surpassed the magnetic energy imposed by the applied field. Thus, the magnetic characteristics of the **Nk@A** can be ascribed to those expected from a soft ferromagnetic material, even at 312K. This kind of ferromagnetic character can be understood from MR imaging which showed high relaxivity values without the interference of Au nanoshell. Moreover, in the case of hyperthermia, the **Nk@A** showed increased heat dissipation under microwave irradiation in a short span of time.

**Figure 5:**
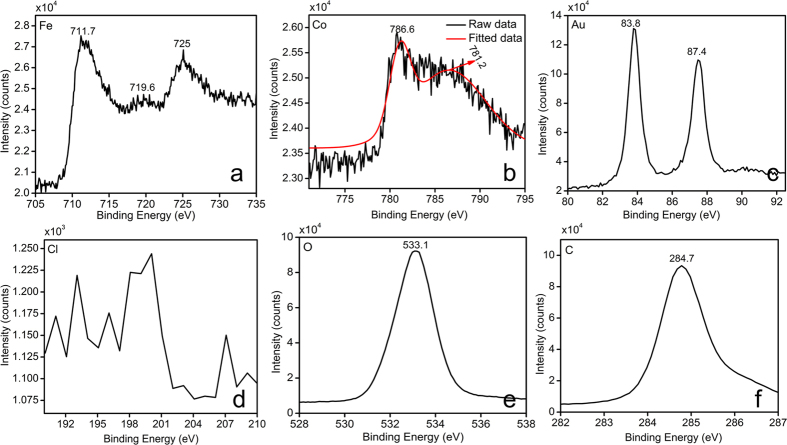
Elemental analysis of **Nk@A** by XPS which clearly shows the presence of elements such as Fe, Co, Au, C, O and absence of Cl.

XPS measurements determined the binding energies and composition of **Nk@A**. The elements viz., Au, Co, Fe, C and O existed within the range from 0 to ≈1300 eV. Core level spectra were recorded and represented in Figure S3a in the †ESI. Fe2p3/2 and 2p1/2 peaks from Fig. 5a situated at around 711.7 and 725 eV respectively, were broadened due to the presence of Fe^3+^ ions in tetrahedral sites^39^,^40^. There is also a satellite peak of Fe^3+^ at 719.6 eV, which confirms the presence of Fe^3+^ 41. The orbitals of Co (Fig. 5b) showed that Co2p3/2 electrons exhibited binding energies at 781.2 eV, which corresponds to ions. This may be due to the substitution of Fe^3+^ ions with Co^2+^ in the tetrahedral site. There is also the existence of a peak at 786.6 eV, which again confirms the presence of Co^2+^. This is in accordance with the XRD data, which proves that there are no mixed phases of CoO or Fe_2_O_3_ in the **Nk** samples^41^. Figure 5c shows Au binding energies with doublet peaks at 83.8 and 87.4eV thus denoting the Au state of Au4f7/2 and Au4f5/2, respectively. This shows that gold ions are completely converted into metallic Au^0^, leading to the formation of **CSNPs**. Interestingly, the spectrum did not show any detectable Cl2p signal^42^ which further proved the complete reduction of Au onto the surface of **Nk** (Fig. 5d). The O1s peak showed in Fig. 5e confirmed the presence of oxygen atoms^43^. The C1s signature markers of carbon atom binding energy at 284.7 eV were taken as a reference (Fig. 5f)^43^. Therefore, XPS pattern was in good agreement with XRD data, TEM-EDS and line scanning results.

### Tethering folic acid linker and Doxorubicin molecules on Nk@A

**Figure 6:**
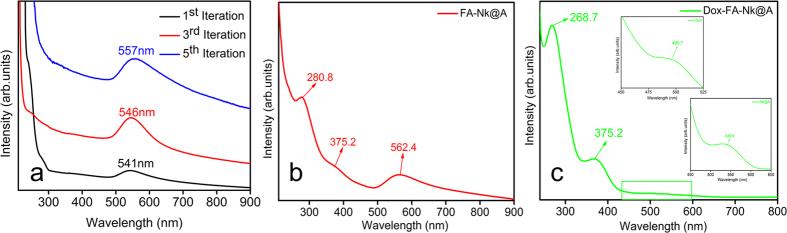
(**a**) UV-Visible absorption spectra showing different iterations from 1–5 with the red shift of Au peak representing the increment of nanoshell, UV-Vis spectra of (**b**) **FA** functionalization, (**c**) binding of **Dox** onto **FA-Nk@A**.

UV-Visible absorption spectra of different iterations of **Nk@A** were performed as seen in Fig. 6a. As the number of gold iterations on **Nk** increased from one to three, there was a bathochromic shift of surface plasmon resonance peak (**SPR**) from 541 to 546 nm. In addition, as the iteration increased from three to five, this **SPR** peak further shifted from 546 to 551 nm. This bathochromic shift in the **SPR** peak was related to the increment in the thickness of gold shells on the surface of the magnetic core^18^. Moreover, as iterations increased, the charge density and the amplitude of the free electron oscillation inside the particles also increased. This may be due to the increased surface coating of CTAB, thus causing enhanced plasmonic absorption^44^. The spectra of **Nk** and Au seeds are also shown in Figure S2 in the †ESI. UV-Visible spectra of **FA** attached **Nk@A** (Fig. 6b) shows distinct peaks at 280.8 and 375.2 nm, which are signature markers of **FA**. Accordingly, there was a bathochromic shift in the **SPR** peak after **FA** attachment which showed a peak at 562.4 nm; this shift confirmed that **FA** formed a complex with **Nk@A**^45^. **Dox** attachment was confirmed from the peak (inset) at 490.7 nm with minor red-shift of **Nk@A** peak (538.9 nm) and slight blue-shift of the **FA** attachment (268.7 & 375.2 nm). These shifts ensured the formation of the **Dox-FA-Nk@A** complex, which is shown in Fig. 6c.

**Figure 7:**
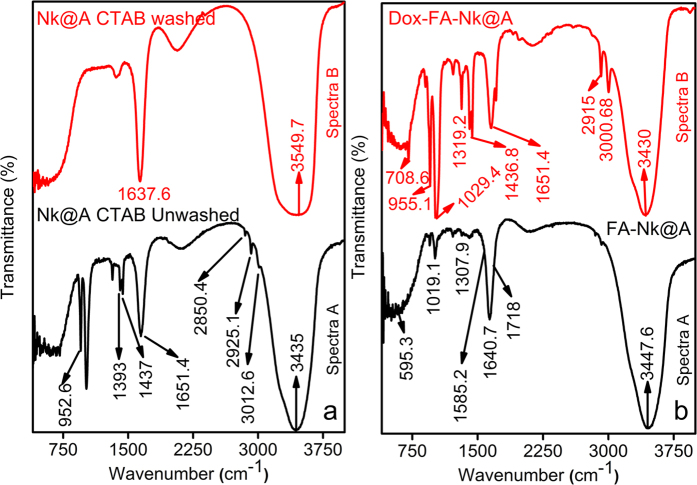
FTIR spectra of (**a**) **Nk@A** with CTAB before and after washing, (**b**) The attachment of **FA** and **Dox** onto **Nk@A.**

FTIR spectra of functional organic markers on the surface of **Nk@A** is shown in Fig. 7a. Spectra A represented only CTAB peaks (Table S2 in the †ESI)^46^, which proved that there was an excess amount of CTAB in the solution. However, spectra B which was analyzed after repeated centrifugation and washing the **Nk@A** solution, showed a minor peak of CTAB which stabilizes the Au shell (Table S2 in the †ESI). This was also demonstrated from its cytocompatibility towards L6 cells, which was evident from MTT assay and confocal microscopy studies.

Figure 7b shows FTIR spectra of **FA-Nk@A** and **Dox-FA-Nk@A**. Spectra A represents both activated **FA**, which show bands at 1640.7 cm^−1^ and 1718 cm^−1^ expressing –CH stretch and –NH stretch, respectively. **FA** conjugation to **Nk@A** was confirmed from –NH and –CH stretch; while the asymmetric stretching of primary amines –NH and bending vibrations of –CO confirmed the formation of amide linkage between **FA** and **Nk@A** at 1585.2 cm^−1^ 47. Spectra B represents the attachment of **Dox** moieties onto **FA-Nk@A**. The interaction between these molecules was via amide linkage, which involved –NH amino group of FA and –COOH carboxylic group of **Dox**. Bands representing these attachments were 1436.8 cm^−1^ that represents anhydride =CO stretch and 1651.4 cm^−1^ denotes amide stretch of =CO. The peak at 2915 cm^−1^ was a classic peak of secondary –NH2 bending, and peak at 3000.68 cm^−1^ corresponded to primary –NH_2_ bending^47^.

TGA analysis was carried out for all samples in the temperature range of 30–900 °C under N_2_ flow atmosphere and any change in % weight loss was recorded. TGA graph (Figure S3b in the †ESI) shows **Nk**, **Nk@A**, **FA-Nk@A**, **Dox-FA-Nk@A**, activated **FA** and **Dox**. Initially, activated **FA** and **Dox** showed a gradual weight loss from 30 to 100 °C. The degradation of these moieties was rapid because they are completely organic in nature. **Nk** showed a rapid degradation at 122 °C, which was due to the complete evaporation of water molecules. Then, there was a solid plot up to 555 °C and a drop at 728 °C followed by a slow degradation of bound chlorides and hydroxides. In the case of **Nk@A**, the initial weight loss from 30 to 288 °C was due to the complete desorption of water and CTAB molecules from the surface^48^. The second degradation, from 289 to 595 °C, was a result of the covalent interaction of CTA^+^ ions; the final degradation at a higher temperature, from 596 to 900 °C was most likely due to the electrostatic interaction of the ammonium group from CTAB attached to the Au **NP**s surface^49^. In **FA-Nk@A** complex, the weight loss in the range of 30–100 °C was due to the desorption of intercrystalline water molecules. The second degradation, in the range of 101–750 °C, was due to **FA** covalent attachment, which was seemingly induced by the disintegration of groups like hydroxyl, carboxyl and amino groups present in **FA**^50^. **Dox-FA-Nk@A** complex initially showed degradation of water moieties and weak surface interactions of hydrogen with the **Nk@A**. The second decomposition from 96 to 740 °C, was due to the decomposition of **FA** and **Dox** from the **NPs** complex and the decomposition from 741 to 900 °C is due to slow degradation of **Nk@A**^51^.

### Intracellular localization of Dox & Dox-FA-Nk@A

**Figure 8:**
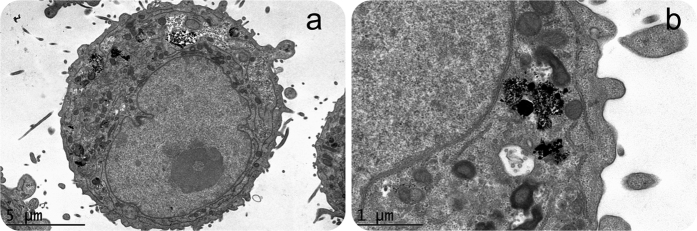
*In-vitro* cellular uptake of **Nk@A** (**a**) CryoTEM image showing the uptake of **Nk@A** by Hep2 cells, (**b**) magnified view of vesicles showing the cluster of **Nk@A** entry by the process of endocytosis.

CryoTEM analysis confirmed **Nk@A** internalization and endocytic cavities that contained **NPs** (Fig. 8a,b) with aggregation, as the pH was acidic. This observation gave an insight towards the mechansim of receptor-mediated endocytosis of **Nk@A**, which can be attributed to **FA** and folate receptor interactions^52^. Moreover, the cellular uptake of **NPs** is also dependent on the surface charge. Zeta potential (ζ) values for **Nk**, **Nk@A**, **FA-Nk@A** and **Dox-FA-Nk@A** complex were in the range of +11.9 to +12.1 mV, +33.4 to +37.7 mV, −35.9 to −38.4 mV, +16.8 to +19.4 mV, respectively (Figure S3c in the †ESI)^53^,^54^,^55^. This charge dependency is due to the stability of the complex, which conciliates the harsh physiological milieu, such as in the bloodstream or inside the cell. The mechanism of cellular uptake for positively charged nanoparticles is interaction of the positive moieties with the negatively charged cell surface^56^. Rotello and coworkers studied the effect of surface charge on the stability of amine functionalized gold nanoparticles. It was found that net positive charge caused more displacement of ligands in extracellular^57^ and intracellular environments^58^.

The tracking of **Dox-FA-Nk@A** inside the cells was performed using confocal microscopy. Hep2 cell lines were incubated only with **Dox** (without any fluorescent dye, since **Dox** itself is a good bioimaging agent) and it showed the internalization. Initially, **Dox** was distributed all over the cytoplasm (Figure S4a in the †ESI) and cellular membrane, but after 24 h of incubation, the **Dox** signal in nucleus was very high due to the migration of **Dox** to this compartment and its release promoted by internal pH changes (Figure S4b in the †ESI). This confirmed that the binding of **Dox** molecules to the nuclei by intercalating into DNA leads to inhibition of macromolecular biosynthesis^59^.

**Figure 9:**
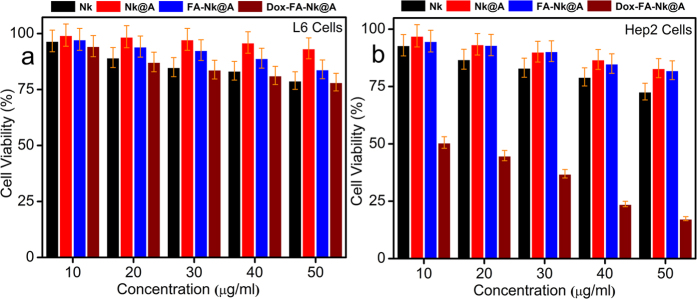
MTT assay of (**a**) L6 cells, (**b**) Hep2 cells with **Nk**, **Nk@A**, **FA-Nk@A** showing no apoptosis even at higher concentrations but **Dox-FA-Nk@A** treated L6 cells showing very negligible cell death and Hep2 cells showing more than 80% cell death at high concentrations.

In order to assess the cell viability, MTT assay was carried out by incubating L6 and Hep2 cells with **Nk**, **Nk@A**, **FA-Nk@A** and **Dox-FA-Nk@A** for 24 h in the concentration range of 10–50 μg/mL (Fig. 9a,b). Even though the concentrations of all the above complexes increased to 50 μg/ml, there was not much cytotoxicity observed for L6 cells. Similarly, Hep2 cells also showed less cytotoxicity when exposed to **Nk**, **Nk@A** and **FA-Nk@A** at concentrations as high as 50 μg/ml. However, the Hep2 cell viability started decreasing rapidly when the concentration of **Dox-FA-Nk@A** was increased from 10 to 50 μg/ml. This may be due to the overexpression of folate receptors on the surface of Hep2 cells, which increases receptor mediated endocytosis and overall cellular uptake^60^.

**Figure 10:**
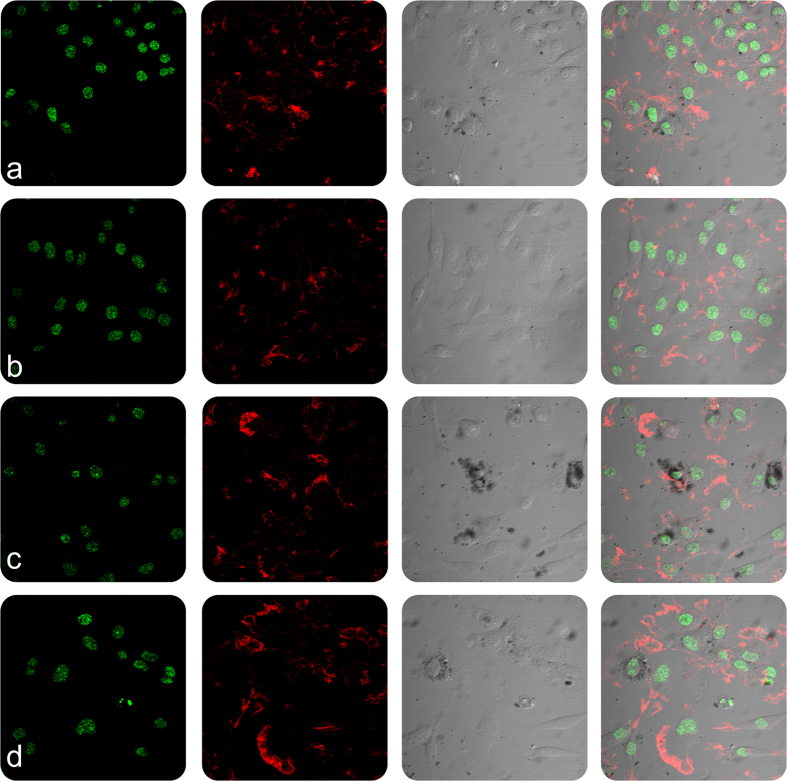
Confocal microscopy study representing the morphology of L6 cells treated with nanoparticles for 24 h (**a**) **Nk**, (**b**) **Nk@A**, (**c**) **FA-Nk@A**, (**d**) **Dox-FA-Nk@A** which shows no cell death even at higher concentration of nanoparticles (Scale bar-20 μm).

**Figure 11:**
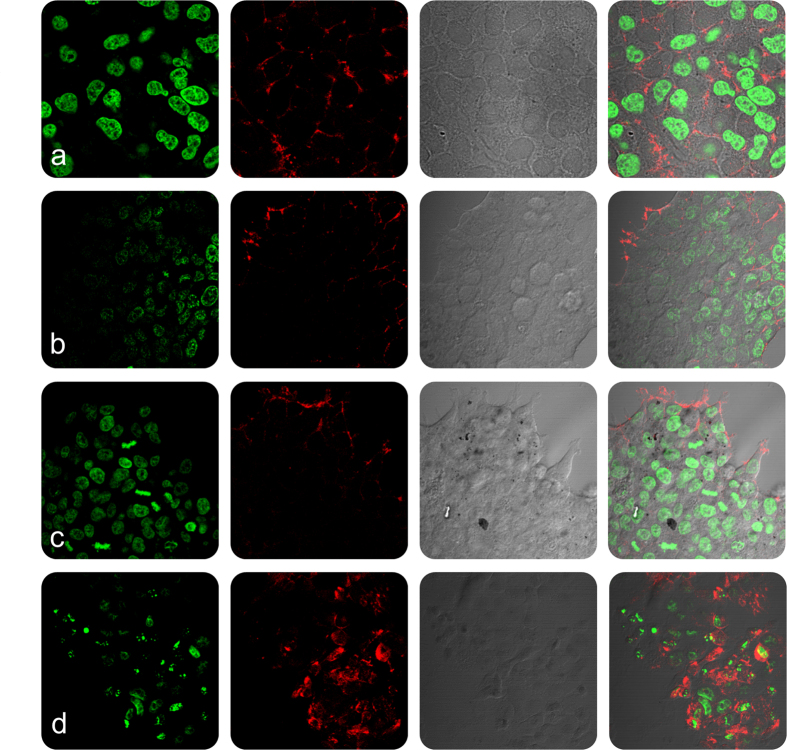
Confocal microscopy study representing the morphology of Hep2 cells treated with nanoparticles for 24 h (**a**) **Nk**, (**b**) **Nk@A**, (**c**) **FA-Nk@A** which shows negligible cell death but (**d**) **Dox-FA-Nk@A** clearly shows the increased cell death at higher concentration of nanoparticles (Scale bar-20 μm).

In order to evaluate the cellular and nuclear morphology of L6 and Hep2 cells on incubation with **Nk**, **Nk@A**, **FA-Nk@A** and **Dox-FA-Nk@A**, the cells were examined using confocal microscopy (Figs 10 and 11). The morphology of cells treated with **Nk**, **Nk@A** and **FA-Nk@A** did not show any noticeable change in both L6 and Hep2 cells. The activity of **Dox-FA-Nk@A**, illustrated in Fig. 10(d), showed no effect on L6 cells, while Hep2 cells [Fig. 11(d)] exhibited major apoptosis caused by disruption of nuclear membrane, which leads to total cell damage. This proved that the **Dox-FA-Nk@A** acted as an efficient nanocargo in delivering the **Dox**, which finally causes cell death. The increased cytotoxicity of **Dox-FA-Nk@A** can be due to the active transport of **Dox** by receptor-mediated endocytosis mechanism as compared to the passive diffusion of free **Dox** into the cells^61^.

### *In-vitro* Dox release kinetics

**Figure 12:**
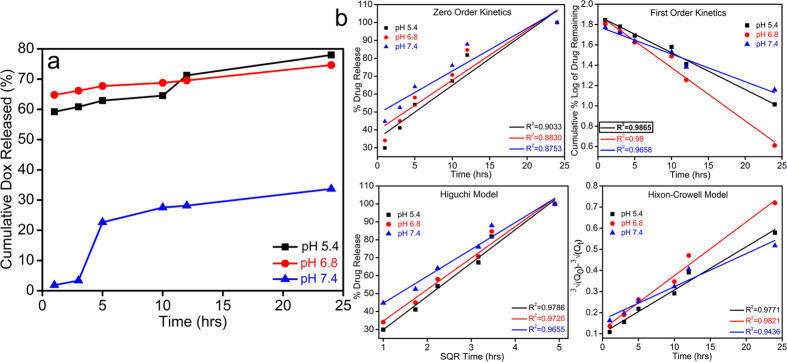
(**a**) Cumulative release of **Dox** at 3 different pH, (**b**) Various plots representing different fitting in kinetic models of drug release.

The *in-vitro* drug release behavior of **Dox-FA-Nk@A** was assessed by using 3 different pH of PBS buffers (5.4, 6.8 & 7.4) for 24 h. We determined that the **NPs** system was both pH dependent and site-specific; which made it as a unique drug delivery system for cancer therapy. As pH decreased in the cellular organelles, thereby increasing the release of drugs as seen in Fig. 12a. The cumulative release of **Dox** at 3 different pH after 24 h was 77.9% at pH 5.4, 74.6% at pH 6.8, and 33.6% at pH 7.4, thus confirming pH-dependent release mechanism. The pH 7.4 mimics normal physiological pH; hence, drug release is minimum. Moreover, the tumor microenvironment exhibits a pH of 6.8^62^,^63^ where drug release is comparatively more, which occurs due to the partial dissociation of the amide bonds between **FA** and **Dox** molecules. The complex showed maximum amount of drug release at pH 5.4. This pH is a signature marker of endosomal acidic pH consequently leading to the dissociation of the drug from the complex by breaking the amide bond between **FA** and **Dox** molecules. Moreover, **Dox** becomes highly water-soluble as well as hydrophilic at lower pH as compared to neutral pH. Hence, **Dox** is in its inactive form in normal tissues at neutral pH, while it gains its activity in cancerous tissue at lower pH^64^.

These results were fitted to different drug kinetic models such as zero order kinetics, first order kinetics, Higuchi model and Hixon-Crowell model (Fig. 12b). The drug release followed first order rate kinetics model which was attributed to the high regression coefficient value (**R**^**2**^ **=** **0.9865**). This confirms that the drug release is pH as well as concentration dependent mechanism.

### Magnetic Resonance Imaging in normal and cancerous cells

**Figure 13:**
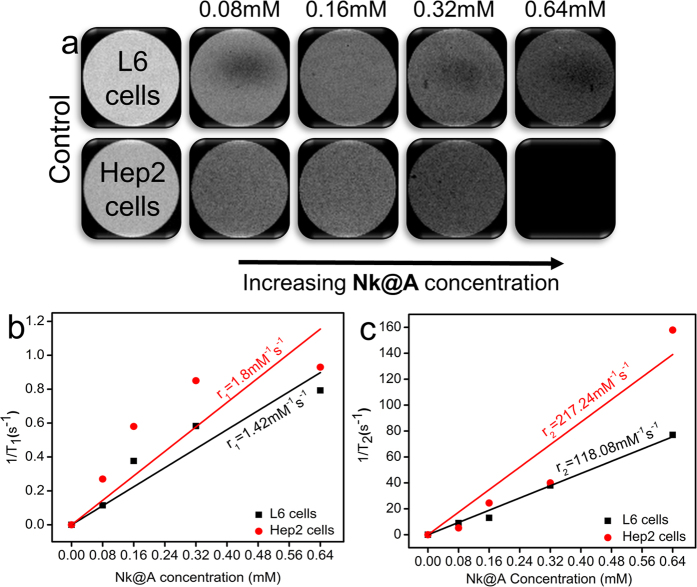
(**a**) T_2_ contrast image of **Nk@A** as the concentration increases the darkening effect also increases depending upon the type of cells, (**b**) r_1_, (**c**) r_2_ relaxivity values of **Nk@A** incubated with L6 and Hep2 cells.

**Nk@A** was employed as an efficient MR imaging contrast agent. **It was** cultured with both L6 and Hep2 cells. The cells were harvested and then resuspended in PBS with agar gel. These agar phantoms were used to evaluate the T_1_ and T_2_-weighted images shown in Fig. 13a (only T_2_-weighted images). As the concentration increased from 0.08–0.64 mM of **Nk@A**, there was an increment in T_2_-weighed image, which became darker with both cells. The linear relationship was calculated in order to obtain the longitudinal relaxivity r_1_ and transverse relaxivity r_2_ of both L6 and Hep2 cells incubated with **Nk@A** (Fig. 13b,c). The r_1_/r_2_ and r_2_/r_1_ values were calculated and are shown in Table S3 in the †ESI. We found that the r_2_/r_1_value of L6 was 83.15 and for Hep2 it was 120.68. This was much higher than r_1_/r_2_ of both, which confirmed that **Nk@A** were efficient T_2_ contrast agent as compared to T_1_. But in the previous report for CoFe_2_O_4_@Au, r_2_/r_1_value was around 33^21^. So, we concluded that the increment in the r_2_/r_1_ value was mainly due to Au iterations.

The r_2_ value of **Nk@A** with L6 was 118.08 mM^−1^s^−1^ and Hep2 was 217.24 mM^−1^s^−1^. This is highly comparable to the clinically used MRI contrast agent such as Feridex 105 mM^−1^s^−1^ 65, which shows noticeable changes after injecting iron oxide **NPs**^66^. Therefore this confirmed that the Au nanoshell around **Nk** did not play a role in **Nk** core spin^67^. But the r_2_ value with Hep2 cells showed significant increment when compared to L6 cells because of gold iterations. The other reason might be the uptake of gold coated magnetic **NPs** by cancerous cells was higher than that of normal cells^68^ because the electrostatic interactions of surface charge from gold coating cause more interaction with the cell^69^. Finally, we demonstrated that the **Nk@A** can be effectively used as an efficient T_2_ contrast agent.

### Microwave based Hyperthermia therapy

**Figure 14:**
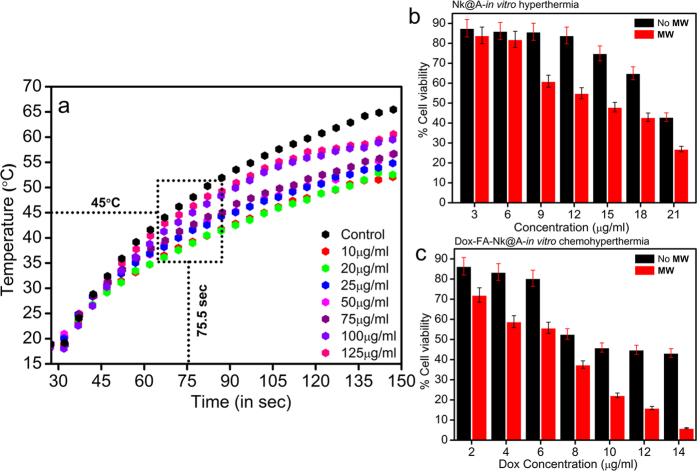
(**a**) Plot showing increment of temperature as a function of time with the increased concentrations of **Nk@A** as a potential hyperthermal agent, MTT assay of Hep2 cells (24 h) subjected to hyperthermia (50 °C for 50 sec): (**b**) *in-vitro* hyperthermia where cells treated with **Nk@A** which represents the killing of cells as the concentration increases by producing enough heat, (**c**) *in-vitro* chemohyperthermia where cells treated with **Dox-FA-Nk@A** which represents the killing as the concentration increases by producing heat and also release of **Dox**.

Hyperthermia therapy involves increase in the temperature of tissues or cells, so that they become more susceptible to anti-cancer drugs. **Nk@A** nanoparticles were tested as a hyperthermal agent under microwave (**MW**) irradiation by using a microcoaxial double slot antenna as an applicator. The increment of temperature as a function of time was measured under ISM (Industrial, Scientific and Medical) approved frequency of 2.45 GHz in order to induce localized hyperthermia. The applicator was inserted in the phosphate buffered saline (PBS) containing **Nk@A** of various concentrations (10–125 μg/ml) and the increment of temperature was measured by using noninterfering fiber optic probes. The **MW** was irradiated using a home-made setup^70^ for 150 sec at 6 W. Interestingly, the temperature increment was very rapid and reached 45 °C in around 75 sec, which was enough to kill cancer cells; the temperature raised upto 50–60 °C within 150 sec for **Nk@A** (Fig. 14a).

The PBS was used as a control which showed maximum rise in temperature as water has the highest absorption of **MW**^71^. This temperature increment was purely based on the Au iterations on **Nks**. Au nanoshell as well as superparamagnetic core led to energy increment which may be due to magnetic anisotropy^72^ as compared to **Nks** in xylene at 2.45 GHz^73^. The temperature increment was much higher when compared with Au@γ-Fe_2_O_3_, which was around 38 °C at 2.45 GHz in water, for 10 mins at a power of 120 W^74^.

### *In-vitro* Hyperthermia and Chemo-Hyperthermia therapy

Chemo-hyperthermal effect or thermo-chemosensitization is quantified by the interaction of anti-cancer drugs with cells at elevated temperature. The thermal enhancement ratio (TER) for **Dox** is 1 at two different temperatures (41.5 °C versus 43.5 °C, respectively)^75^. There is a relationship between drug-heat interactions on cell cytotoxicity. This pharmacodynamics is responsible for enhanced killing of cells. The mechanism of thermal enhancement for drug cytotoxicity includes enhanced drug uptake as well as DNA damage and retardation of DNA repair^33^. There are previous studies, which reported the thermal enhancement of cellular cytotoxicity when drug interaction with cells takes place at higher temperatures^76^. In this report, an increase in **Nk@A** concentration shows an increased cell mortality in addition to **MW** radiation exposure for 50 sec. The cells interacting with increased **Nk@A** concentration, but without any exposure to **MW** irradiation (Fig. 14b), showed comparatively lesser mortality than the exposed ones. Both Au shell and superparamagnetic core absorb **MW**, due to which hyperthermia is induced in the cells, thus consequently leading to cell death. The viability of Hep2 cells was suppressed more by **Dox-FA-Nk@A** when exposed to microwave as compared to unexposed ones (Fig. 14c). The **Dox** concentration was considered based on its half-maximal inhibitory concentration (IC50) value of 12.5 μg. Hence, here the **Dox** tethered **Nk@A** concentration was in the range of 2–14 μg/ml. The IC50 value of **Dox-FA-Nk@A-MW** was 8 μg as compared to the IC50 value of **Dox-FA-Nk@A**, which is 12 μg. This clearly shows thermal enhancement of **Dox** cytotoxicity at lower IC50 values in comparison with **Dox** alone. Hence, **Dox** orchestrated **Nk@A** efficiently inhibited the cell viability at elevated temperatures and very low concentrations of **Dox**, thus improving the therapeutic proficiency along with minimum side-effects. Concurrently, this combined therapy shows efficient synergism, thus exhibiting inimical effect on Hep2 cells.

In summary, we have developed **SPR** enhanced **Nk@A** by multiple iterative method, which proved to be a promising nanomaterial with multifunctional properties specifically in the field of cancer nanotheranostics. These multiple iterations provided a new platform for high surface functionalization. This helped in the efficient delivery of drugs, following first order rate kinetics. This **Nk@A** was also used as a competent MRI contrast agent and proved to be an effective T_2_ agent with high relaxivity values in the presence of both L6 and Hep2 cells. Finally, **Nk@A** was used as a hyperthermal agent. In an *in-vitro* study using Hep2 cells, both **Nk@A** and **Dox-FA-Nk@A** on exposure to microwave irradiation using 2.45 GHz for 50 sec showed cell mortality. It was found that there is an enhancement of cell mortality when **MW** based hyperthermia collates with Chemotherapy. With this efficiency, **Nk@A** can be used for potential applications as a single nanomaterial for 3 different uses, from tracking, diagnosing to therapeutics.

## Materials and Methods

### Materials

Ferric (III) chloride (FeCl_3_•6H_2_O, 97%), Cobalt (II) nitrate hexahydrate (CoN_2_O_6_•6H_2_O, 99.999%), sodium hydroxide (NaOH, >98%), Gold (III) chloride trihydrate (HAuCl_4_.3H_2_O, ≥99.9%), L-Ascorbic acid (AA) (C_6_H_8_O_6_, ≥99.0%), Dimethyl sulfoxide (DMSO) (CH_3_SOCH_3_, ≥99.9%), Sodium chloride (NaCl, ≥99.5%), Hexadecyltrimethylammonium bromide (CTAB) (CH_3_(CH_2_)_15_N(Br)(CH_3_)_3_, ≥99.0%), Folic acid (**FA**) (C_19_H_19_N_7_O_6_, ≥97%), N-Hydroxysuccinimide (NHS) (C_4_H_5_NO_3_, 98%), N,N′-Dicyclohexylcarbodiimide (DCC) (C_6_H_11_N=C=NC_6_H_11_, 99%), Triethylamine (TEA) ((C_2_H_5_)_3_N, ≥99%), Doxorubicin hydrochloride (**Dox**) (C_27_H_29_NO_11_•HCl), MTT [3-(4,5-dimethylthiazol-2-yl) 2,5-diphenyltetrazolium bromide], Hoechst Stain solution, Phalloidin–Tetramethylrhodamine B isothiocyanate, and ethanol (CH_3_CH_2_OH, ≥99.8%) were purchased from Sigma-Aldrich (Mexico). Dulbecco’s Modified Eagle Medium (DMEM), fetal bovine serum (FBS), and streptomycin were obtained from Gibco, Life Technologies. Agarose (UltraPure, Agarose) was purchased from Invitrogen, Thermo scientific. Deionized water (DI) was used for all experiments. All chemicals were used directly without any further purification.

### Synthesis of Cobalt ferrite (Nk) and gold-coated Nk (Nk@A) NPs

#### Nk preparation

Magnetic **Nk** were synthesized using co-precipitation method. The precursors 0.5 M of ferric (III) chloride and 0.25 M of cobalt (II) nitrate hexahydrate were taken in the ratio of 1:0.5 and in order to avoid the precipitation of the salts, they were initially dissolved separately in 10 ml of nitrogen (N_2_) degassed DI water and mixed with 1.5 M solution of 40 ml NaOH, which was used as a reducing agent. The entire synthesis process was carried out under N_2_ atmosphere for 1.5 h at 80 °C (pH 12). The black resultant precipitate was separated using a strong magnet and it was washed 3 times with DI water. This pure **Nk** were further used for **Nk@A** formation.

#### Nk seed preparation

200 μl of synthesized **Nk** was centrifuged and dispersed in 1 ml of DI water (pH adjusted to 7). Then, 400 μl of DMSO was added and the mixture was stirred continuously under N_2_ atmosphere at 75 °C for 3 h to reduce the aggregation of **NPs**.

#### Preparation of gold seed solution

Au coating was carried out for 5 iterations, 5 aliquots of gold seed solution were prepared freshly by mixing 0.5 ml (1 M) of CTAB, 1 ml (50 mM) of ascorbic acid, and 100 μl (1M) of HAuCl_4_ solution. This solution complex mixture was sonicated for 15 mins. The golden yellow color immediately changed to a milky orange color and then to a milky white color (Figure S1b, (b*) in the †ESI).

#### Synthesis of Nk@A CSNPs

The seed solution of **Nk** and gold were used to synthesize **Nk@A**. In this case, the ratio of 1:5 was taken. This was because we had already optimized the ratio with 1:5, 1:7, and 1:9 and found that 1:5 was appropriate for the **Nk@A**. Initially, 1 part of milky white colored gold seed solution was added dropwise to the faint brown-colored **Nk** seed solution. This solution mixture was stirred for 2 h continuously until the brown colored solution turned into purple. Then, the gold iteration was continued for 5 times for every 2 h. Finally, a dark purple color **Nk@A** solution was obtained. Then, these **Nk@A** were magnetically separated (after each iteration) by magnetic separation technique and they were washed twice with a mixture of hexane and ethanol to obtain high purity **NPs** excluding excess gold **NPs**. The washed **NPs** were centrifuged again twice to remove excess CTAB from the solution.

#### FA Activation and attachment to Nk@A

Activated **FA** was used for the attachment onto the surface of **Nk@A**. The reason behind the activation of **FA** was to activate the carboxylate group. This was carried out by dissolving **FA** (0.25 g) into 20 ml of DMSO which was subjected for 1 h sonication to ensure complete dispersion. Later, carboxylate group present in **FA** was activated by mixing 0.125 gm of DCC and 0.225 gm of NHS. The complete reaction was carried out under N_2_ atmosphere at 30 °C for 12 h (**FA**/DCC/NHS molar ratio 2:1:2). The resultant product was filtered using Whatman filter paper; then it was further used for attach onto **Nk@A**.

Activated **FA** was used to attach onto the **Nk@A**. This attachment was carried out by mixing 1ml of activated **FA** and 10 ml of **Nk@A** under N_2_ atmosphere and stirring the mixture continuously for 5 h. Then, N_2_ atmosphere was detached and the reaction was continuously stirred for 24 h. Finally, this reaction mixture was filtered using Whatman filter paper. Then the process of dialysis was carried out to eliminate unreacted **FA** using a 3000 kDa dialysis membrane in PBS (pH 7.4). After centrifugation, the pellet was again dialyzed in DI water for a period of 24 h. The activated **FA** binding onto the surface of **Nk@A** was studied using UV-Vis spectroscopy analysis.

#### Synthesis of Dox-FA-Nk@A CSNPs complex

Anthracycline antibiotic **Dox** was used to kill cancerous cells using **FA-Nk@A**. Activated **FA** functionalization on **Nk@A** acted as an anchor for the binding of **Dox**. To bind **Dox**, 5 ml **FA-Nk@A** was mixed with 1ml of TEA and ml of DMSO as a solvent; finally, 400μl of 2.36 mM **Dox** solution were added. This mixture was purged using N_2_ gas under continuous stirring at 60 °C for 5 h. The final complex solution was dialyzed to remove the unbound or excess of **Dox**. The **Dox** binding was studied by characterization techniques, like FTIR and UV-Vis spectroscopy.

#### Dox loading efficiency and *in-vitro* Dox release

Before initiating the drug release studies, it was very significant to determine the **Dox** loading efficiency because the exact amount of **Dox** bound onto **FA-Nk@A** complex was so decisive to calculate the proper drug delivery study (in the †ESI). **Dox** release studies were carried out by dialysis process. Drug release study was determined at 3 different PBS at 37 °C in an incubator of pH 5.3, 6.8 and 7.4 with continuous stirring to simulate the intercellular, intracellular and external environment of cancer cells. To carry out this study, 2 ml of dialyzed **Dox-FA-Nk@A** complex was used. Then, 1ml of sample was withdrawn from the system every 40mins to determine the drug content. To compensate the PBS solution as soon as 1ml was drawn, it was replaced with the same equivalent volume. The amount of **Dox** released was determined using UV-Vis spectrophotometer at 485nm which was the signature absorbance of **Dox**. All the experiments were repeated thrice for all the samples. This drug release study was also explained with different drug kinetics models in order to explain the release mechanism.

### Characterization

#### UV-Vis spectroscopy

UV-Vis spectra was obtained using Shimadzu Corporation UV-2401PC UV-Vis spectrometer.

#### X-Ray diffraction (XRD) measurement

The crystallinity of the **Nk** and **Nk@A** were recorded by using X’Pert PRO XRD spectrometer (PANalytical B.V., Holland) from 10 to 80° (2θ value) using Cu K-α radiation (0.15418 nm).

#### Fourier transform infrared (FT-IR) spectroscopy

FT-IR spectra were obtained on a Nicolet iS50 FT-IR Spectrometer (Thermo Scientific).

#### Thermogravimetric analysis (TGA)

Thermal analysis was carried out for liquid samples using a TGA Q50 (TA Instruments) from 30 to 900 °C under nitrogen flow with a heating rate of 10 °C/min.

#### High Resolution Transmission electron microscopy (HRTEM)

TEM images were taken on a HRTEM (JEOL, JEM-ARM200F) equipped with HAADF-STEM (high-angle annular dark-field scanning transmission electron microscopy) detector and Oxford XMax 80 Energy Dispersive X-Ray Spectrometer (EDS). The sample was prepared in such a way that aggregation in the grid was avoided. Then, 10 μl of sample were dispersed in 100 μl of isopropanol, which was sonicated for 30 mins; then a drop of sonicated nanoparticle dispersion was placed onto the amorphous carbon-coated 200 mesh copper grid (Ted Pella, Inc.). Finally, the sample was allowed to dry at ambient temperature before it was loaded into the microscope.

#### CryoTEM

The internalization of **NPs** inside the cells was confirmed by cryoTEM analysis. 5 μl of **Nk@A** nanoparticle suspensions were made into a thin liquid film which was prepared on lacy carbon grid (Pelco, USA) and then quenched into liquid ethane to freeze the sample using a Leica EM-CPC chamber. Tecnai F20 (FEI) operated at 200 kV to obtain the images which were recorded with a USC1000 slow scan CCD camera (Gatan) at 50000x.

#### X-Ray photoelectron spectroscopy (XPS) Analysis

XPS analysis were performed using a K-Alpha X ray Photoelectron Spectrometer (XPS) System (Surface Analysis, Thermo Scientific). Monochromated, Micro-focused Al Kα was used as an X-ray source type. The binding energy of C1s carbon at 284.7 eV was used for calibration.

#### Magnetic measurements

**Nk** and **Nk@A** magnetic properties were measured using a superconducting quantum interference device (SQUID), (Quantum Design, MPMS3). The magnetization hysteresis of the samples was obtained by changing H between +70 to −70 kOe at 3 different temperatures, like 5 K, 300 K and 312 K. The hysteresis of the samples was also obtained at 100 kOe using a temperature interval from 1.8 to 312 K.

#### Zeta potential measurements

The zeta potential values were determined by using a Zetasizer Nano ZS90 (Malvern instruments) at 25 °C at a wavelength of He-Ne laser 633 nm, Max 4 mW at a scattering angle of 90° using a Universal ‘dip’ cell kit. Data were obtained using a monomodal acquisition according to the Smoluchowski theory. The measurements were repeated 3 times. Before the analysis the samples were well sonicated for 1 h to avoid aggregation.

#### Magnetic Resonance Imaging (MRI) experiments

MR imaging was performed with a 7T clinical Signa HDxt scanner (Varian). T_2_-weighted images were acquired using the following parameters: 7T, Repetition time TR = 2000 ms, fast spin echo, FOV = 3*3 cm, Echo time TE = 15–250 ms, slice thickness = 4 mm and resolution 256 × 256 points. For T_1_ measurements, coronal spin-echo sequences with fixed echo time (TE) = 24 ms and varying repetition time (TR) (25 ms to 4 s) were used. **Nk@A** suspensions was taken at varying concentrations.

#### Microwave (**MW**) experiment setup

The setup used to apply the **MW** electromagnetic field to perform the experiments consisted of a generator (SML 03, Rhode & Schwarz, Germany) set to a frequency of 2.45 GHz. This signal was then amplified using an RF & MW module power amplifier (1164-BBM3Q6AHM, Empower, USA). The output power was then monitored through the use of a dual direct coupler (DC7154M, Amplifier Research, USA) and a power meter (PM2002, Amplifier Research, USA) to ensure an output power of 6W and to monitor the reflected power of the system. To adjust the standing wave ratio (SWR) to an optimal value, a coaxial stub tuner (1878C, Maury Microwave Corp., USA) was used along a network analyzer (E5071B, Agilent Technologies, USA) to measure and reduce the SWR to a minimum prior to each experiment.

#### Temperature Measurements

Non electromagnetic interfering optical fiber probes temperature sensors (M3300, Luxtron, USA) were used to record temperature increment. The temperature was measured inside the PBS. Each test lasted 200 sec in order to study the temperature response as a function of time. The temperatures were recorded every second during the experiments using True Temp software (Luxtron, USA).

#### Confocal Imaging

Z-series confocal images were collected using Zeiss LSM 700 confocal microscope fitted with a 40X oil-immersion lens. Images were processed using Zen 2012 software.

All the above analysis and characterization techniques used liquid sample (FTIR, TGA, UV-Vis spec, HRTEM, Zeta potential, Flow cytometry analysis), powder form (SQUID), and thin film (XRD).

## Additional Information

**How to cite this article**: Ravichandran, M. *et al.* Plasmonic/Magnetic Multifunctional nanoplatform for Cancer Theranostics. *Sci. Rep.* **6**, 34874; doi: 10.1038/srep34874 (2016).

## Supplementary Material

Supplementary Information

## Figures and Tables

**Figure 1:**
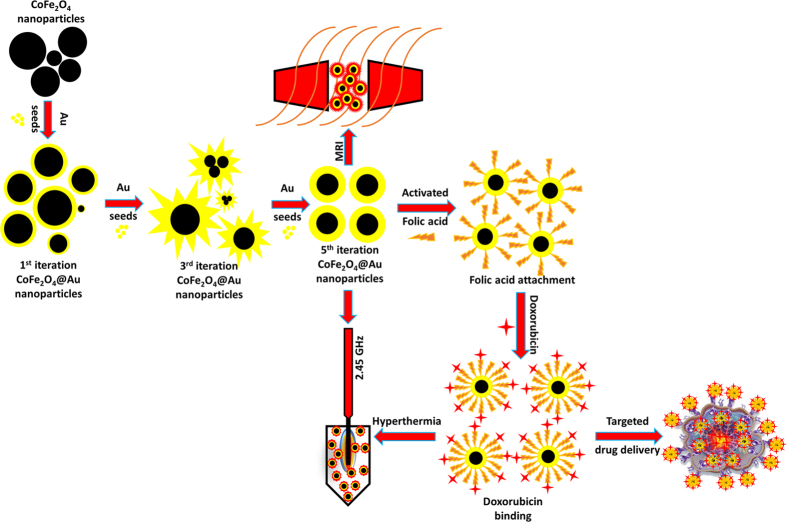
Schematic representation of CoFe_2_O_4_ synthesis and Au iterations from 1–5 to enhance the **SPR** property. The surface is modified by attaching activated folic acid for specific targeting of cancer cells and also binding with anti-cancer drug doxorubicin for efficient chemotherapy. Finally, the whole complex is employed as MRI contrast agent and microwave based hyperthermal cargo at 2.45 GHz to track and treat cancer cells with high localization.
