# Supplementary material for: Plasmonic/Magnetic Multifunctional nanoplatform for Cancer Theranostics
Source: Sci Rep. 2016 Oct 10;6:34874. doi: 10.1038/srep34874 (PMC5056510; doi:10.1038/srep34874)
Supplement: Supplementary Information [file srep34874-s1.doc]

Electronic Supplementary Information

**Plasmonic/Magnetic Multifunctional nanoplatform for Cancer Theranostics**

M. Ravichandran^a^, Goldie Oza^b^, S. Velumani^c§^, Jose Tapia Ramirez^b§^, Francisco Garcia-Sierra^d^, Norma Barragan Andrade^d^, A. Vera^e^, L. Leija^e^ and Marco A. Garza-Navarro^f^

^a^Program on Nanoscience and Nanotechnology,

^b^Department of Genetics and Molecular Biology,

^c^Department of Electrical Engineering,

^d^Department of Cell Biology,

^e^Department of Electrical Engineering - Bioelectronics Section, CINVESTAV-IPN, Av. 2508 National Polytechnic Institute, Gustavo A. Madero, San Pedro Zacatenco, 07360 Mexico City.

^f^Department of Mechanical and Electrical Engineering, Universidad Autonoma de Nuevo Leon, San Nicolás de Los Garza, Nuevo León, 66451 Mexico city.

^§^Correspondence and requests for materials should be addressed to: S.V. ([velu@cinvestav.mx](mailto:velu@cinvestav.mx)), J.T.R. ([jtapia@cinvestav.mx](mailto:jtapia@cinvestav.mx))

*Cell Labeling:* Confocal microscopy was employed to visualize the cellular and nuclear morphology caused by bare **Nk**, **Nk@A**, **FA-Nk@A** and especially **Dox-FA-Nk@A**. Both L6 and Hep2 were seeded on 13 mm glass coverslips positioned inside a 12-well culture plate with a seeding density of 1000 cells/coverslip. After 24h, the adherent cells were washed with PBS and media was replaced and incubated for 24h at 37°C. To remove the loosely bound and free **NPs** the cells were washed with PBS. Using 2% paraformaldehyde the cells are fixed. After washing with the cells are permeabilized with 500µl of PBS-triton 0.1% and again washed with PBS. Then the cells are stained with 300µl of Hoechst of 1:4000 dilution in 1x PBS. Later again stained with 300µl of Phalloidin/Rhodamine dye. Finally after washing, vectashield was used to seal the coverslip on the glass slide to observe under confocal microscopy.

*Cell Culture:* L6 (skeletal muscle cell line) and Hep2 cells (laryngeal carcinoma cell line) were cultured in DMEM supplemented with 100UmL^-1^ penicillin, 10% FBS and 100µgmL^-1^ streptomycin, with a 5% CO_2_ atmosphere in a humidified incubator at 37°C.

*In-Vitro Cytotoxicity Assay:* MTT assay was carried out to determine the cell viability. L6 and Hep2 cells were seeded into a 24-well plate with a cell density of 1×10^5^ cells/plate and suspended in DMEM for 24h at 37°C in a 5% CO_2_ atmosphere. Later, the medium was replaced containing different concentrations of **Nk**, **Nk@A**, **FA-Nk@A** and especially **Dox-FA-Nk@A** in the range of 10-50 µg/mL. Then plates were placed at 37°C in a humidified 5% CO_2_ incubator and after 24h MTT-assay was performed.

For MTT assay, 10μL of MTT (5mg/ml) in DMEM was added to each well and incubated for 3h under same conditions. After incubation, removal of MTT solution was done and DMSO (100μL) was added to dissolve formazan crystals. The plates were kept on a shaker for 10 min and then absorbance was recorded at 490nm using a micro plate reader (Thermo Electron Corporation, USA). The absorbance was recorded in triplicate and cell viability was calculated from their average values.

*Hyperthermia measurements:* The heating effect of **Nk@A** was studied using **MW** based hyperthermia at 2.45GHz. The aqueous sample of various **Nk@A** concentrations was taken and well dispersed with PBS (volume is made upto 1ml). Then the applicator was placed in the sample and the temperature is measured. The samples were exposed to **MW** for 150sec at 6W.

*In-Vitro hyperthermia and chemo-hyperthermia:* To assess the *in-vitro* hyperthermia and chemo-hyperthermia, Hep2 cells were used with a cell density of 1×10^5^ cells/plate grown using DMEM medium. Once the DMEM medium is replaced, the cells are incubated with **Nk@A** and **Dox-FA-Nk@A**. Then the cells are incubated for 3h and washed with PBS to remove excess **NPs**. Later the cells are detached from the plate by employing the method of trypsinization and transferred to the centrifuge tube with 1ml of DMEM. Two sets of samples were taken, one for control without exposing to **MW** and another for hyperthermia testing. The tube containing cells with the **NPs** were placed in a water bath maintaining temperature of 37°C and exposed to microwave for 50°±0.5°C for 50 sec, where the temperature was measured by fiber optics temperature probe. After the experiment, the cells are placed in an incubator for 24h and MTT assay has been carried out.

For *in-vitro* hyperthermia and chemo-hyperthermia studies the concentration of the **NPs** were chosen by considering the inhibitory concentration (IC50) of **Dox** in the control samples and the **Dox** drug concentration was fixed to 12.5µg/ml for further studies.

*Phantom preparation for MRI*: A pellet containing **Nk** and **Nk@A** labelled with L6 and Hep2 cells was mixed with 1.5ml of preheated 0.5% agarose gel at 40°C. Then the labeled cells are incorporated with agar phantom which is cooled down to room temperature to solidify and stored at 4°C. The agar gel with the cells are considered as a control for phantom MR imaging studies.

***Dox*** *loading efficiency calculation:*

Concentration of drug initially loaded = 1.4285mM

Concentration of unbound drug = 0.0705mM (Abs-0.474)

[Concentration of drug is calculated using the standard calibration curve of **Dox** (Straight line equation: y = 6.721x)]

Concentration of drug loaded = Concentration of drug initially loaded – Concentration of drug of unbound drug

=1.4285mM – 0.0705mM

= 1.358mM

Drug Loading Efficiency (%) = (Concentration of drug finally loaded / Concentration of drug initially loaded)*100

= 1.358/1.428*100

= 95.09%


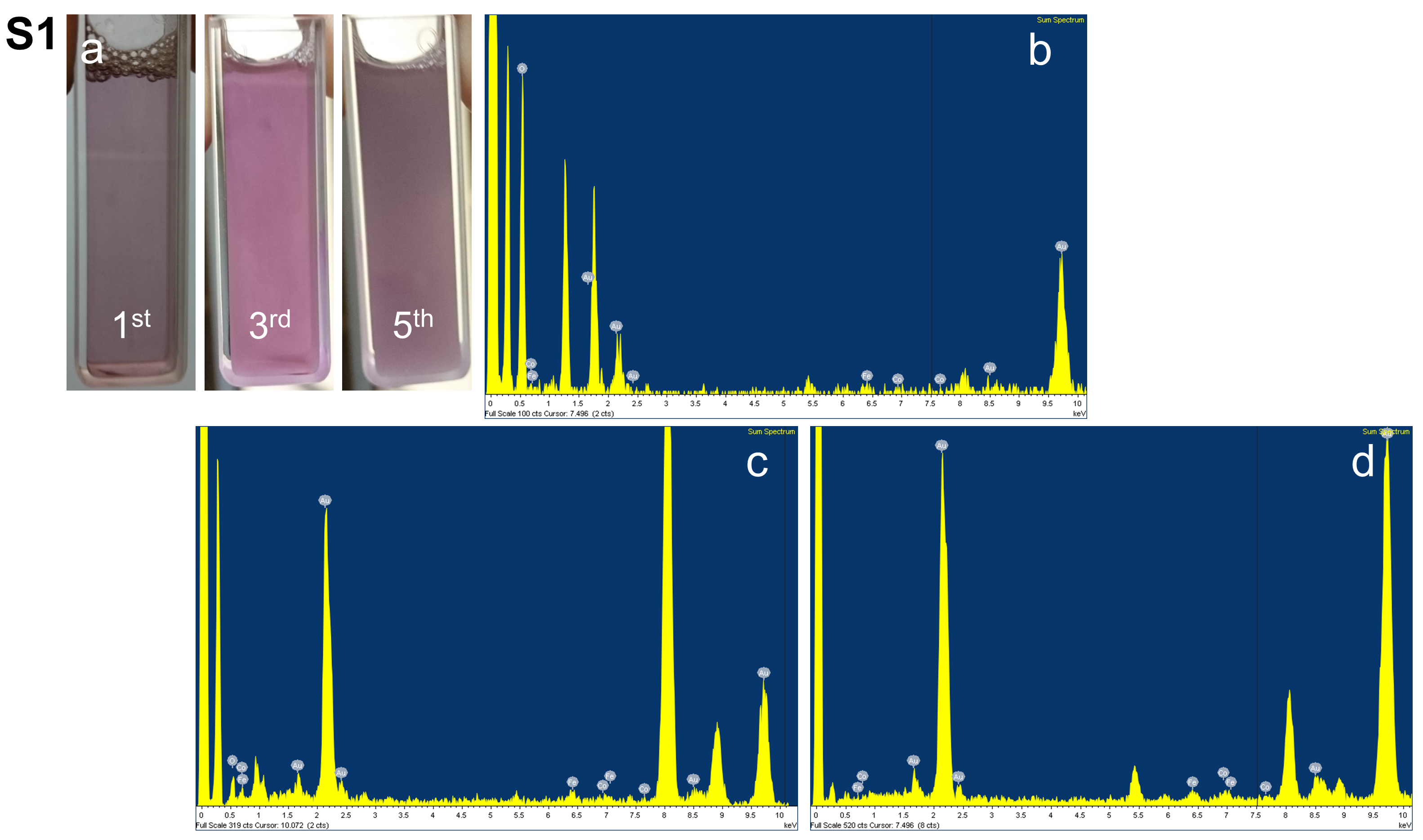


**Figure S1.** a) Colour of the **Nk@A** solution at 1^st^ iteration, 3^rd^ iteration and 5^th^ iteration which changes from light purple to dark purple, EDS analysis of various Au iterations such as b) 1^st^ iteration, c) 3^rd^ iteration, d)5^th^ iteration.

**
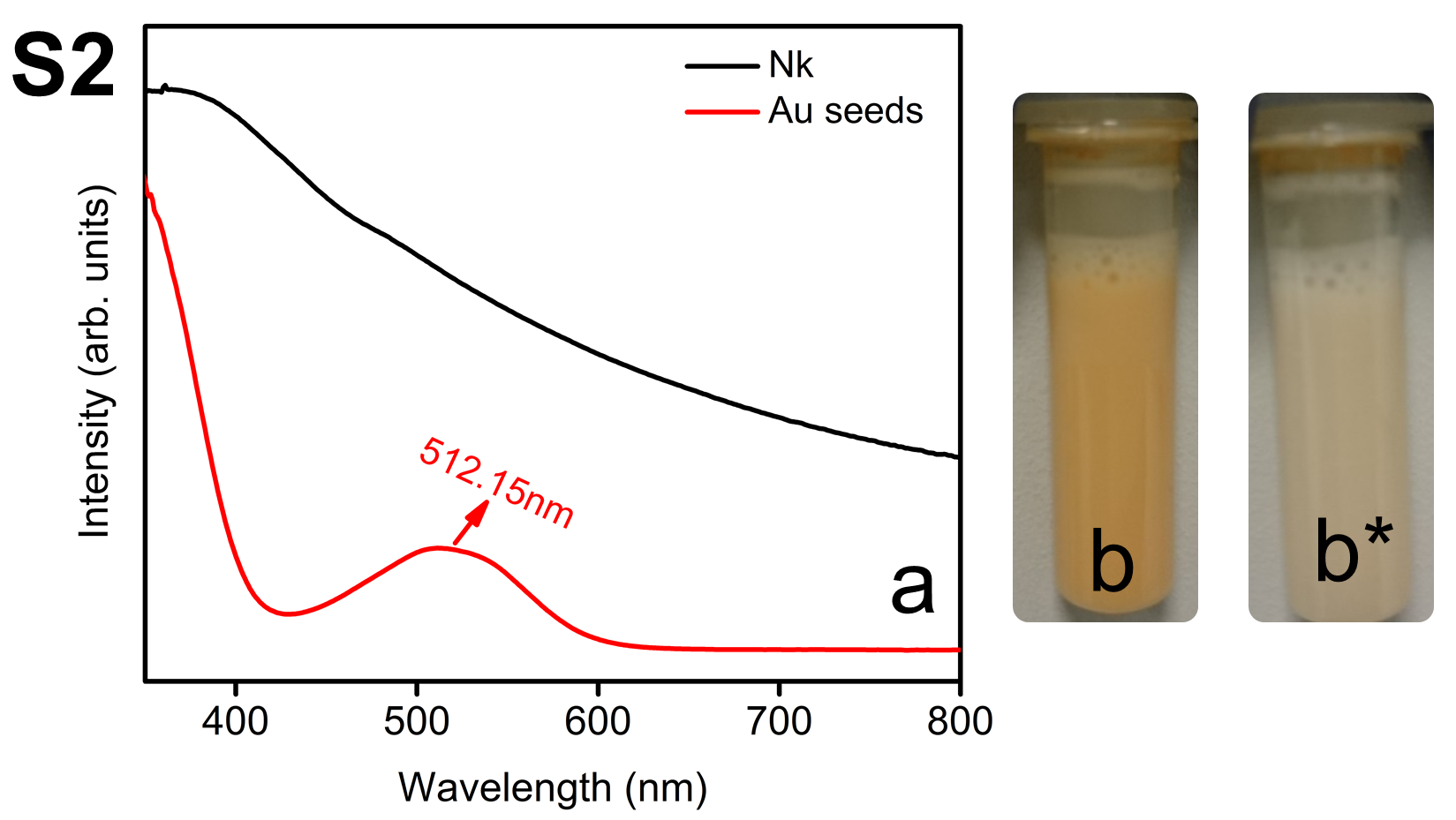
**

**Figure S2.** a) UV-Vis spectra of **Nk** and Au seeds solution which is a combination of Au precursor, CTAB and AA exhibiting a **SPR** peak at 512.15nm, Gold seed solution b) initially milky orange colour b*) later changed to milky white colour.

**
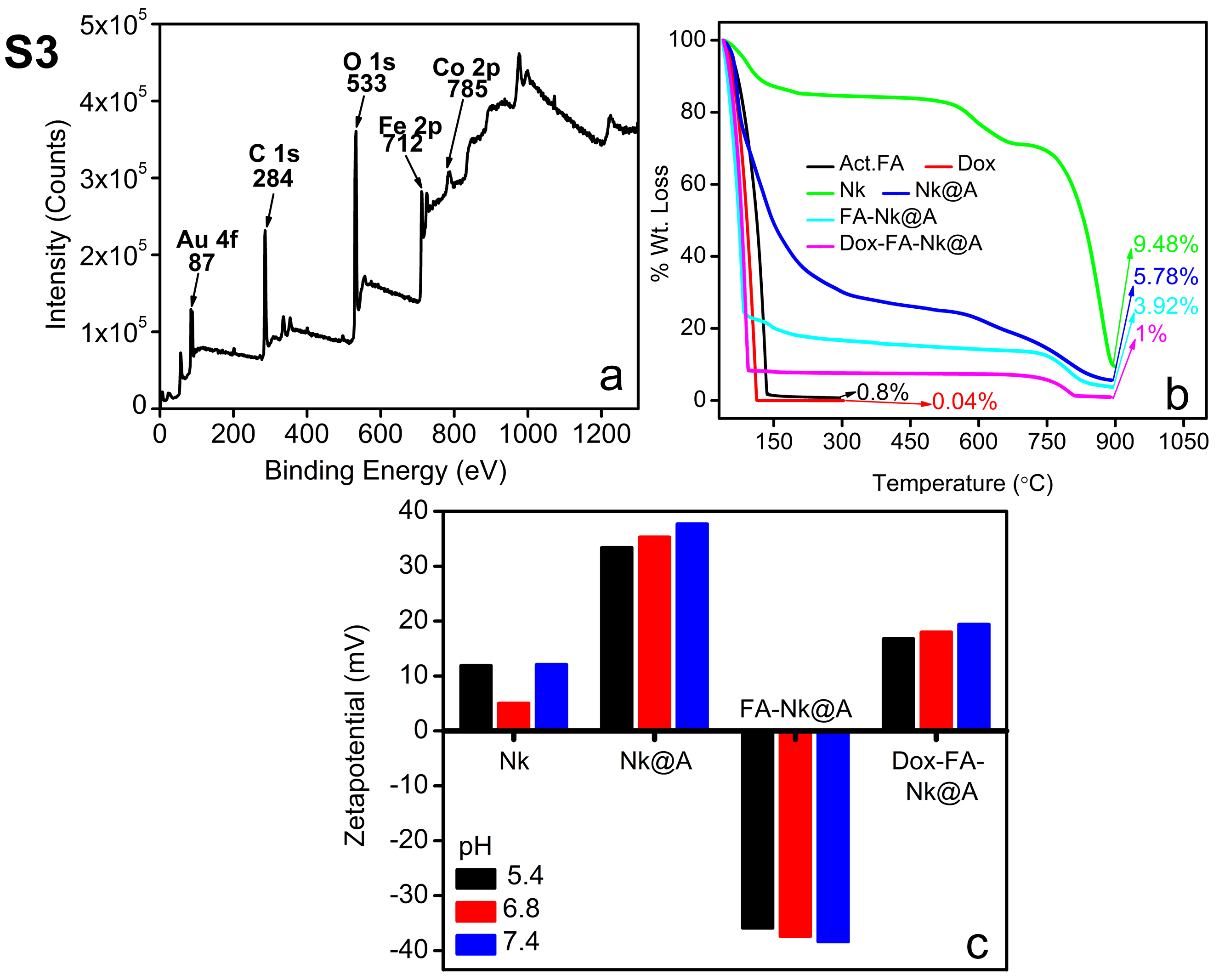
**

**Figure S3.** a) Scan survey of XPS showing the presence of all the elements, b) TGA analysis of all complexes to study the stability, c) Zeta potential of **Nk, Nk@A, FA** functionalized **Nk@A** and **Dox** binding onto **FA-Nk@A.**


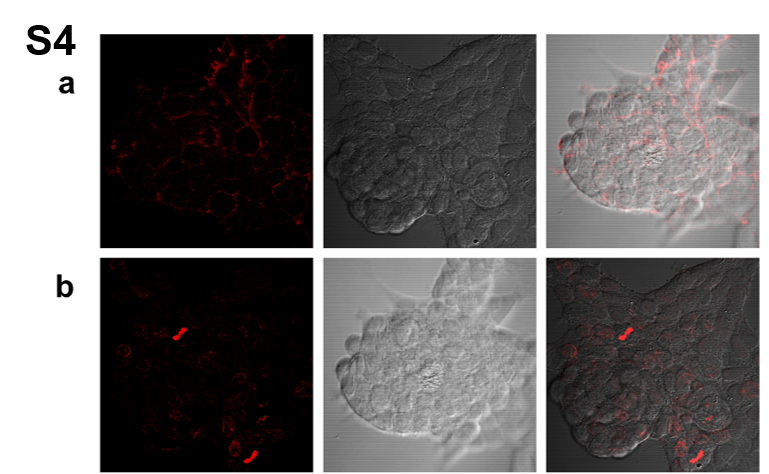


**Figure S4.** Confocal microscopy image representing the internalization of **Dox** moieties at various time periods a) initial (0h), b) final (24h).

**Table S1.** SQUID measurements of **Nk@A** showing Hc, Mr & Ms at 5K, 300K & 312K.

| **Nk@A** | Hc (Oe) | Mr (emu/g) | Ms (emu/g) |
| --- | --- | --- | --- |
| 5K | -145.84 | 7.53 | 74.03 |
| 300K | -7.42 | 0.39 | 46.72 |
| 312K | -25.38 | 0.24 | 45.39 |

**Table S2.** FTIR peaks of **Nk@A** with CTAB before and after washing.

| Sample | Peak(cm^-1^) | Functional groups |
| --- | --- | --- |
| **Nk@A** sample unwashed  (Spectra A) | 952.6 | N–H bending mode |
|  | 1651.4, 1437, 1393 | deformation of –CH and –CH2 groups |
|  | 3012.6, 2925.1, 2850.4 | stretching vibrations of –CH3 and –CH2 groups |
|  | 3435 | N–H stretching vibration |
| **Nk@A** sample washed  (Spectra B) | 1637.6 | scissor bending vibration of H_2_O molecules |
|  | 3549.7 | O–H stretching in H-bonded water |

**Table S3.** Calculation of relaxivity values.

| **Nk@A** incubated with | L6 cells | Hep2 cells |
| --- | --- | --- |
| r_1_/r_2_ | 0.0120 | 0.0143 |
| r_2_/r_1_ | 83.15 | 120.68 |
